# The Relations between 3-year Changes in Physical Fitness and Academic Performance in Nationally Representative Sample of Junior High School Students

**DOI:** 10.1038/s41598-018-34370-2

**Published:** 2018-10-29

**Authors:** Shu-Shih Hsieh, Jia-Ren Tsai, Shao-Hsi Chang, Chih-Fu Cheng, Yao-Ting Sung, Tsung-Min Hung

**Affiliations:** 1 Department of Physical Education, National Taiwan Normal University, Taipei, Taiwan; 2 Department of Statistics and Information Science, Fu Jen Catholic University, New Taipei, Taiwan; 3 Department of Educational Psychology and Counseling, National Taiwan Normal University, Taipei, Taiwan; 4 Institute for Research Excellence in Learning Science, National Taiwan Normal University, Taipei, Taiwan

## Abstract

The objective of the current study was to examine the relationship between different components of physical fitness across 3 years of junior high school with academic performance assessed at the end of the period. Two nationwide representative datasets were used. The first was the physical fitness profile assessed at the beginning of each of the three school years. The second contained the scores on a standardized test administered at the end of the third year. All data were standardized by calculating percentile rank (PR). Students were classified as “*High-fit*” if their fitness scores ≧ top 25% PR on the age- and sex-adjusted norms. All other students were classified as “*not high-fit*”. The relationships between fitness and exam performance were tested adjusting for sex, body mass index, and level of urbanization. Students who were in the high-fit group in both years 1 and 3 academically outperformed those who were outside this classification during both assessments. The degree of outperformance was greatest for those who were aerobically fit, followed by those who were high-fit in terms of muscular endurance, muscular strength, and flexibility, respectively. It is therefore concluded that the relationship between physical fitness and academic performance in Taiwanese junior high school students is strongest in the case of aerobic fitness.

## Introduction

Evidence has supported the role of physical activity, either via designed school-based programs^1,2^ or activities during school recess, in improving cognitive function^1^ and academic performance^1,2^. Studies have indicated the role of fitness in mediating the association between physical activity and academic achievement via higher levels of fitness^3^. The relationship between fitness, cognition, and academic success in children is not surprising given accumulating evidence about the positive influence of fitness on the maturity of brain areas responsible for higher-order cognitions and learning, including the prefrontal cortex and the hippocampus^4^. Indeed, previous studies suggest the existence of a long-term association between overall physical fitness, usually consisting of performance on a variety of component fitness dimensions such as aerobic fitness, muscular fitness, and flexibility, and academic performance. For example, Bezold *et al*.^5^ and London and Castrechini^6^ found that students who maintained or improved overall physical fitness over time had better academic performance. However, there is little evidence on whether individual fitness measures correlate differently with academic performance.

Previous findings regarding the role of individual fitness measures have been inconclusive. While cross-sectional studies have found strong evidence for a positive relationship between academic performance and aerobic fitness, the results in relation to muscular fitness or flexibility have been unclear^7^. Findings from longitudinal studies have also been equivocal. For example, studies^8–11^ have found that students who maintained high levels of aerobic fitness had superior academic achievement relative to those who did not. Chen *et al*.^12^ confirmed this finding in the case of aerobic fitness, but reported that muscular endurance and flexibility were not associated with academic achievement. On the other hand, Liao *et al*.^13^ reported that muscular strength and flexibility were, in fact, positively associated with academic performance. It is possible that the discrepancy in results stems from differences in data collection and methods used to measure fitness or academic outcomes. For example, while some data were obtained from regional locations (i.e., Taichung^12^, East Central Illinois^9^, West Virginia^8^) others derived from national samples (i.e., Taiwan)^13^. Some studies^8–11^ used the PACER (Progressive Aerobic Cardiovascular Endurance Run) test m to measure fitness, while others used standardized national fitness tests^12,13^. There were also differences in the measures of academic outcomes. Some studies used grades^11,12^ or teachers’ ratings^10^ whereas others used standardized tests^8,9,13^.

The objective of the current study then was to examine the association of a broad range of physical fitness measures, including aerobic fitness, muscular endurance, muscular strength, and flexibility, over 3 years of junior high school with academic performance assessed at the end of this period using a nationally representative population. Based upon previous findings, it was hypothesized that students with high-level or improved fitness over 3 years would have better academic achievement at the end of the period, and that aerobic fitness would have the strongest relationship with academic achievement, following by muscular fitness (endurance and strength) and flexibility.

## Methods

### Participants and Procedures

Data on a total of 398,870 junior high school students between the ages of 12 to 15 (Mean_year 1_ = 12.8, SD_year 1_ = 0.5; Mean_year 3_ = 14.8, SD_year 3_ = 0.5) were collected from 5 cohorts - those attending junior high schools during 2006–2009, 2007–2010, 2008–2011, 2009–2012, and 2010–2013. For all students, academic performance was assessed by the BCTJH, which was administered at the end of the final year. The retrieval of data from students was conducted on October 2014 and was approved by the ethics review panel of the Ministry of Education in Taiwan (MOE). No informed consent form from students was required from the ethics review panel because all data was de-identified. Data collection took place from September of 2006 to May of 2013. The current study made use of two nationally representative datasets. The first dataset contained a physical fitness profile of junior high school students collected by the Ministry of Education in Taiwan (MOE). Every junior high school student is required to take these tests within the first 4 weeks of each academic year (which starts in September) over the 3-year period. The second dataset contained the scores on the Basic Competence Test for Junior High School Students (BCTJH), a compulsory examination administered by the MOE at the end of junior high school (at the end of May) to all high-school-bound students.

Students’ physical fitness and BCTJH scores were matched on their name and personal identification number. Data from high-school-bound juniors who took the BCTJH in 2009, 2010, 2011, 2012, and 2013 in their third year were examined, with fitness scores obtained at the beginning of the first and third year being matched with BCTHJ scores at the end of the third year. For example, students who sat for the BCTHJ in May of 2013 had their test results matched with their fitness scores from September, 2010 and September, 2012. Analogous procedures were applied to students who sat for the BCTJH in the other four years (i.e., 2009, 2010, 2011, and 2012).

### Measurements

#### Physical Fitness

Four components of fitness were assessed, including aerobic fitness, muscular endurance, muscular strength, and flexibility, by physical education (P.E.) instructors. Aerobic fitness was assessed by a 1600- and 800-m run test for boys and girls, respectively. This test is a standard measure of aerobic fitness used for Taiwanese students from elementary to college level, and has been shown to be a valid measure with high mean criterion-related validity (*r* = 0.79)^14^. Students were instructed to give his/her best effort to run/walk the distance as fast as possible. The score on this test was the total time in seconds to cover the 1600- or 800-m distance, with shorter time indicating better performance.

Muscular endurance was measured by a 1-minute bent-leg curl-ups test. This test is a standard measure of muscular endurance used for Taiwanese students from elementary to college level, and has been commonly used in previous research^12,13^. Students were instructed to lay with their backs on a cushion, with their feet flat on the floor, and knees bent at 90°. Students crossed their arms over their chests so that their hands touched the opposite shoulder. The trunk was then raised in an attempt to touch the elbows to the patellae. The score on this test was the total number of correctly performed (both elbows touching both knees) curl-ups within 1 minute, with higher counts indicates better abdominal muscular endurance.

Muscular strength was assessed by the distance obtained in a standing long-jump test. This test has been widely used^15,16^ and has been shown to be a reliable measure of muscular strength (intra-class coefficient = 0.93)^17^. Students were instructed to stand behind the starting line with feet hip-width apart and knees bent. They swung their arms as they jumped, and the distance jumped was measured from the starting line to the heel landing closest to it. If the students fell backward, the distance was measured from the starting line to the body part closest to it. Longer distance indicated better performance.

Flexibility was assessed with a sit-and-reach test which measured the distance from the fingertips to the edge of a ruler in centimeters. As a measure of flexibility, this test has been shown to have a mean criterion-related validity of 0.67^18^. Students were instructed to remove his/her shoes and sit on the floor with legs stretched straight out. The soles of the feet were placed flat against the vertical ‘sit-and-reach’ ruler and both arms were extended forward along a measuring line as far as possible, with the palms facing down. Measurements were taken when the participants had held the extended position for at least two seconds. Further distances indicated better lower back and hip joint flexibility.

The raw scores for each fitness component in boys and girls were further transformed into the age- and sex-adjusted percentile ranks (PR) in the MOE norms. By doing so, the fitness scores can be standardized and the confounding effects from sex dimorphic and biological maturation on fitness can be ameliorated.

#### Academic Achievement

The BCTJH is a compulsory, nationwide examination given to all high-school-bound students in Taiwan. This measure is not only a standardized and validated measure of academic achievement, students are also highly motivated to do their best when taking the exam since the results will determine their chances of being admitted to competitive high schools which, in turn, will affect their future educational and career path^19,20^. The BCTJH consists of 6 subjects: language (Chinese), foreign language (English), mathematics, social science, science, and an essay. The first five tests consist of computer-scored, multiple choice questions, with each test having a different number of questions. Scale scores ranging from 1 to 80 points are determined for each test based on performance on the questions answer correctly. The essay, which was marked by trained MOE examiners, required that students write down their thoughts on a given topic. The essay had a maximum score of 12 with only even number scores being given (i.e. 2, 4, 6, 8, 10, or 12) with the vast majority of students being given a score of 6 or 8). Thus, the maximum obtainable score on the BCTJH was 412 points. In order to standardize students’ performance, PRs were also computed for each student based on the percentile ranking of their total scores relative to all exam takers in Taiwan. PR is a key factor in the assignment of students to high schools (each high school in Taiwan sets specific entry criteria expressed in term of PR, only accepting students whose PR meet or exceed this). In general, a single PR increase is sufficient to allow entry to a school 1 to 2 place higher in the national rankings.

#### Demographic Characteristics

Data were collected on sex and level of urbanization, which classified students into 3 groups (high, medium, and low urbanization) based on the following parameters: 1) population density; 2) average educational level; 3) percentage of citizens over 65 years old); 4) percentage of the population engaged in agricultural work; and 5) ratio of the number of physicians to the total population. In addition, body mass index (BMI) was obtained by measuring height and weight as a surrogate measure of body composition. BMI of students were classified as underweight, normal, overweight, or obese based upon the age- and sex-adjusted model provided by the MOE.

### Data Analysis

Data on 16,611 students whose fitness scores were more than ±3 SD from the mean at either or both of the two time points were excluded from analysis. These students included those with either very low (n = 7,774) or very high fitness scores (n = 8,837). These extreme cases may have reflected instances where students did not comply with the testing procedures or data input errors. After the exclusion of these students, data remained from 382,259 students.

Levels of aerobic fitness, muscular endurance, muscular strength, and flexibility in the seventh and ninth grades were classified as “*high-fit (H)*” if the raw scores were in the top 25% PR as determined by the age- and sex-adjusted MOE norms, and classified as “*not high-fit (N)*” otherwise. The current study chose the top 25% PR as the cut-off point to truly reflect the idea of “*being fit*”.

Four groups were defined based on fitness levels in the first and third years of junior high school: N-N: students with scores lower than the top 25% PR in both year 1 and year 3; N-H: students with fitness scores lower than the top 25% PR in year 1 but whose fitness was in the top 25% PR in year 3; H-H: students with fitness scores higher in the top 25% in both year 1 and year 3; and H-N: students in the top 25% PR in year 1 who dropped below this level in year 3. This four group comparison design was well suited to determining how high-fit students both differed from others in terms of their exam scores and gauging the practical significance of this in terms of PR.

Data were analyzed using SPSS 21.0, with an alpha of 0.05 set as the threshold of statistical significance. Pearson product-moment correlation coefficients were conducted between demographic variables (i.e., sex, BMI, level of urbanization) and exam scores. Next, two one-way analyses of variance (ANOVAs) were separately performed with *Group* as the between-subjects factor and total score in the BCTJH as the dependent variable. The initial analysis tested the relationship between fitness groups and exam scores with no adjustments made for possible confounding variables, while the second ANOVA included demographic variables shown to be significantly correlated with exam scores as covariates. *Bonferroni*-corrected *t*-tests were utilized for post hoc analyses. Partial eta square (η_p_^2^) and Cohen’s *d* effect sizes were reported to complement the use of significant testing, with the following conventions to determine the magnitude of the effect sizes: 0.01–0.06–0.14 for η_p_^2^, and 0.2–0.5–0.8 for Cohen’s *d* to represent small, medium, and large effect sizes respectively^21^.

## Results

Descriptive statistics for students included at the year 1 and year 3 assessments are presented in Table 1. In addition, Table 2 summarizes students’ year 1 and year 3 fitness scores stratified by sex.

**Table 1:** Descriptive Characteristics at Year 1 and Year 3 Assessments.

Variables	Year 1 (N = 382,259)	Year 3 (N = 382,259)
Age (years)	12.8 (0.5)	14.8 (0.5)
Sex		
*Boys* (%)	51.6	51.6
*Girls* (%)	48.4	48.4
BMI type		
*Obese* (%)	10.1	9.8
*Overweight* (%)	11.8	11.3
*Normal* (%)	60.2	59.3
*Underweight* (%)	17.9	19.7
Level of urbanization		
*High* (%)	48.5	48.5
*Medium* (%)	40.5	40.5
*Low* (%)	11.0	11.0

**Table 2:** Summary of Fitness Scores at Year 1 and Year 3 Assessments.

Fitness components	Boys		Girls	
	Year 1	Year 3	Year 1	Year 3
Aerobic fitness (sec)*	576.5 (108.2)	532.6 (99.5)	293.5 (58.0)	294.6 (56.0)
Muscular endurance (rep)	35.7 (9.0)	41.1 (9.1)	29.5 (8.2)	31.5 (8.6)
Muscular strength (cm)	176.6 (26.6)	200.9 (28.4)	146.6 (23.3)	149.0 (24.0)
Flexibility (cm)	26.1 (8.4)	27.4 (9.5)	30.7 (9.0)	32.1 (9.7)

*Lower value from baseline indicates improved performance.

### Bivariate Correlations

Table 3 summarizes the results of the Pearson product-moment correlations. The results showed that all demographic variables, including sex (*r* = −0.02), BMI type (*r* = −0.06), and level of urbanization (*r* = 0.04) were correlated with exam scores. Thus, all these demographic variables were included as covariates in the following analyses.

**Table 3:** Bivariate Correlations between Demographic Variables and Exam Scores.

Demographic variables	*r*	*p*
BCTJH score		
Sex	−0.02	<0.001
BMI type	−0.06	<0.001
Level of urbanization	0.04	<0.001

### Fitness groups and Academic Performance

Table 4 presents the summary of the results from the crude (unadjusted for covariates) and adjusted models depicting the relationship between different fitness components and exam performance (including raw scores and PR).

**Table 4:** Summary of Exam Performance as a Function of Fitness Groups.

			*F*		*p*		η_p_^2^		Post-hoc	
Groups	Total score	PR	Model 1	Model 2	Model 1	Model 2	Model 1	Model 2	Model 1	Model 2
**Aerobic fitness**			938.3	794.2	<0.05	<0.05	0.007	0.006	1 > 2 > 3 > 4	1 > 2 > 3 > 4
1) H-H group (n = 49226)	286.8 (95.8)	59 (28)								
2) N-H group (n = 43107)	278.8 (95.6)	56 (28)								
3) H-N group (n = 47767)	271.9 (95.7)	54 (28)								
4) N-N group (n = 242159)	264.2 (94.9)	52 (27)								
**Muscular endurance**			865.6	827.7	<0.05	<0.05	0.007	0.006	1 > 2 > 3 = 4	1 > 2 > 3 = 4
1) H-H group (n = 55045)	285.2 (95.0)	58 (28)								
2) N-H group (n = 63931)	277.2 (95.2)	56 (27)								
3) H-N group (n = 31716)	264.2 (97.2)	52 (28)								
4) N-N group (n = 231567)	264.7 (95.0)	52 (27)								
**Muscular strength**			484.0	402.9	<0.05	<0.05	0.004	0.003	1 > 2 > 3 > 4	1 > 2 > 3 > 4
1) H-H group (n = 64409)	280.8 (95.7)	57 (28)								
2) N-H group (n = 46475)	274.7 (96.1)	55 (28)								
3) H-N group (n = 35361)	270.6 (95.9)	54 (28)								
4) N-N group (n = 236014)	265.6 (95.0)	52 (27)								
**Flexibility**			431.5	466.6	<0.05	<0.05	0.003	0.004	1 > 3 > 2 > 4	1 > 3 > 2 > 4
1) H-H group (n = 70573)	281.0 (93.2)	57 (27)								
2) N-H group (n = 38864)	268.3 (96.5)	53 (28)								
3) H-N group (n = 37127)	270.8 (95.8)	54 (28)								
4) N-N group (n = 235695)	266.4 (95.7)	52 (27)								

The inclusion of covariates slightly decreased the strength of the relationship between fitness components and exam scores (Table 4) except in the case of flexibility where the relationship controlling for covariates actually strengthened.

Aerobic fitness was significantly associated with exam scores, *F*(3,382252) = 794.20; *p* < 0.05; η_p_^2^ = 0.006). Post hoc comparisons showed that the H-H group (286.8 ± 95.8) had higher exam scores than the N-H (278.8 ± 95.6; *d* = 0.08), the H-N (271.9 ± 95.7; *d* = 0.16), and the N-N (264.2 ± 94.9; *d* = 0.24) groups. Comparisons between the N-H, the H-N, and the N-N groups indicated that scores in the NI-HF group were higher than that in the H-N (*d* = 0.07) and the N-N group (*d* = 0.15), with a further difference between the latter two (*d* = 0.08).

Muscular endurance was also associated with exam scores, *F*(3,382252) = 827.71; *p* < 0.05; η_p_^2^ = 0.006). Again, the H-H group (285.2 ± 95.0) outscored the N-H (277.2 ± 95.2; *d* = 0.08), the H-N (264.2 ± 97.2; *d* = 0.22), and the N-N (264.7 ± 95.0; *d* = 0.22) groups. The N-H group further outperformed the H-N (*d* = 0.14) and the N-N group (*d* = 0.13). However, there was no difference between the last two.

The muscular strength fitness component was significantly associated with academic performance, *F*(3,382252) = 402.89; *p* < 0.05; η_p_^2^ = 0.003), with the H-H group (280.8 ± 95.7) outperforming the N-H (274.7 ± 96.1; *d* = 0.06), the H-N (270.6 ± 95.9; *d* = 0.11), and the N-N (265.6 ± 95.0; *d* = 0.16) group. Further comparisons showed that the NI-HF group had higher exam scores than the H-N (*d* = 0.04) and the N-N group (*d* = 0.10), with an additional difference between the latter two (*d* = 0.05).

Results for flexibility were quite different from the other three components. Specifically, while this component was significantly related to exam performance, *F*(3,382252) = 466.62; *p* < 0.05; η_p_^2^ = 0.004), and the H-H group (281.0 ± 93.2) had higher scores compared with the H-N (270.8 ± 95.8; *d* = 0.11), N-H (268.3 ± 96.5; *d* = 0.14), and N-N (266.4 ± 95.7; *d* = 0.15) groups, comparisons between the latter three showing that the H-N group had better exam performance than the N-H (*d* = 0.03) and the N-N group (*d* = 0.05), with there being an additional difference between the N-H and the N-N group (*d* = 0.02).

## Discussion

The current study examined the association of physical fitness assessed at the first and third year of junior high school with academic performance measured at the end of the period. Novel aspects of the present study included the use of a broad range of measures in physical fitness, namely aerobic fitness, muscular endurance, muscular strength, and flexibility, and the use of a nationally representative sample of Taiwanese junior high school students from both urban and suburban areas from all regions throughout the country. In addition, the use of the BCTJH, a well standardized examination in which students were strongly motivated to do well in, ensured that there was a very high quality measure of academic performance.

Results indicated that while all components of fitness were positively associated with exam performance, the relationship was strongest for aerobic fitness and weakest for muscular strength and flexibility. The NI-NI groups performed least well on the BCTJH (with the exception of muscular endurance, where this group tied for worst performance). Furthermore, differences in total scores between the H-H group and the N-N group were 23, 20, 15, and 15 for aerobic fitness, muscular endurance, muscular strength, and flexibility, respectively (Table 4). These differences in total scores corresponded to differences in PR of 7, 6, 5, and 5 for aerobic fitness, muscular endurance, muscular strength, and flexibility (Table 4), respectively. Although significant, the partial eta square effect sizes as well as the pair-wise *d* effect sizes were small, reflecting the large sample size. Nevertheless, schools throughout Taiwan are ranked in terms of the PR entry criteria that they set for incoming students. As such, even a single unit PR increase in a student’s exam result is generally enough to allow entry to a school 1 to 2 places higher in the national rankings. This suggest that the effect of better fitness on exam results may be sufficient to allow students to attend a significantly better high school than they would otherwise have been offered entry to.

Our findings are in keeping with previous research employing multiple measures of physical fitness (i.e., aerobic fitness, upper limb strength, lower limb strength) which found that students who maintained a high level of physical fitness also had higher grade point averages over the 4 years of secondary school^22^. This study however is the first to use a large nationwide representative sample.

The present study supports findings from previous research involving similarly aged students^8–12^ regarding the long-term influence of aerobic fitness to academic performance, and is the first to show that prior aerobic fitness has a larger effect on academic scores than other components of fitness. Most previous research had only demonstrated the relationship in cross-sectional studies^7^. Results of the present study further suggest a dose-response relationship during the junior high school period, as indicated by the fact that the H-H group, who presumably were the most aerobically active, had the highest exam scores, followed by the N-H, the H-N, and the N-N groups. The difference between these groups in terms of exam results, corresponding to between 2 and 7 percentiles, may well have been large enough to significantly affect students’ subsequent assignment to high schools.

Regarding muscular endurance, Liao *et al*.^13^ using the curl-ups test demonstrated that muscular endurance was positively associated with a composite score of academic performance in Taiwanese high school students. The current study extended these results to a younger cohort, and went one step further by showing how academic performance varied as a function of changes in muscular endurance over time. Although the effect sizes were small, it was shown that the H-H group outperformed the N-H group by an amount (2 PR) that was big enough to result in an admission to a high school ranked 2 to 4 places higher. Nevertheless, the H-N group did no better on academic performance than the N-N group. These two groups’ results were, on average, 4 to 6 PR lower than H-H and N-H groups.

It is noteworthy that previous studies of muscular fitness exclusively focused on muscular endurance^7^ or a composite measure of muscular fitness^7,23^. However, muscular endurance only accounts for one aspect of muscular fitness, and the current finding is the first to distinguish between muscular endurance and strength in a sample of junior high-school students. While there were similarities in the relationship between these two dimensions with academic performance, one difference was seen in the case of the H-N group. Specifically, the H-N group outperformed the N-N group by a small but possibly meaningful difference (1 PR). The findings regarding muscular endurance and strength collectively suggest that while these two dimensions are both related to academic performance, this relationship may be a result of different underlying mechanisms.

Several studies^24,25^ have previously demonstrated that flexibility was related to academic performance. The current study adds to this finding by providing data on flexibility over 3 years. Results indicated that the H-H group had better academic performance than the other three groups. Once again, the increase in exam scores (up to 5 PR) was large enough to result in noticeable changes to the ranking of high school offering admission. On the other hand, the H-N, the N-H, and the N-N group differed from each other by only a modest amount (1 PR).

Regarding mechanisms underlying the association between fitness and academic performance, executive function, self-regulation, and general life skills may be possible candidates. Both cross-sectional^15^ and longitudinal studies^26^ have reported a mediating role of executive function in the relationship between fitness and academic achievements. Executive function is a top-down, multifaceted cognitive process involving in goal-oriented behaviors^27^. The inhibition and working memory aspects of executive function may be particular relevant to success in subjects such as spelling and math^15,28–30^. Indeed, executive function is not only predictive of successful achievements in school^28–30^ but also especially sensitive to physical activity and its related outcomes (e.g., fitness)^1,31,32^.

The relationship between fitness and academic achievements may also be mediated by self-regulation. Earlier studies have found improved self-regulation following participation in a 3-month school-based physical activity program^33^. Improvement in self-regulation may be associated with better classroom engagement, including better ability to cooperate with teachers and other students, retain relevant information in mind to answer questions, inhibiting maladaptive behaviors, and following instructions and rules^34^, which may, in turn, result in higher academic achievements.

Alternatively, general life skills, including competence (i.e., a positive view of one’s actions in domain specific areas), confidence (i.e., an internal sense of overall positive self-worth and self-efficacy), connection (i.e., positive bonds with people and institutions), character (i.e., an individual’s respect for societal and cultural rules), and caring/compassion (i.e., a person’s sense of sympathy and empathy for others)^35^, might be better in students with superior scholastic performance, and thus they are able to manage their time more effectively, understand the benefits of physical activity, or commit to physical activity on a regular basis.

There are several limitations of this study that should be acknowledged. First, the experimental design employed does not allow definitive causal inferences to be made. Regarding the direction of effects, it remains unclear whether fitness driving academic performance or vice versa (i.e., high achievers in academics are also high achievers in physical fitness tests).

Second, there was a long time lag between year 3 measures in physical fitness and final academic outcomes. It is possible that individual differences in developmental trajectory might confound the data. Similarly, given the absence of year 1 academic outcome data, the current study cannot rule out the confounding effect of individual differences in baseline academic performance.

Third, the current study only focused on the “*quantitative*” aspect of fitness, other skill-oriented aspects, such as object control and motor coordination, were omitted. It is plausible that the “*qualitative*” aspects also account for changes in academic achievements^26^.

Fourth, no measure of socioeconomic status (SES) was taken and thus it was not possible to directly control for this variable^36^. Nevertheless, the inclusion of ‘level of urbanization’ as a covariate may have gone some way towards achieving this, since its definition included several variables (e.g. educational level, percentage of the population engaged in agricultural work) which are strongly linked to SES.

Notably, due to the large sample size, it was possible for even very weak effects to reach statistical significance, and in some cases the partial eta square and pair-wise effect sizes were very small. Some estimate of the practical significance of the findings is conveyed by the reporting of results in terms of PR, but it is hard to generalize this measure to other countries where PR plays a less central role in determining high school placement.

Likewise, the fitness norms established by the MOE were based on relative performance within the population and may not be compatible with criteria based fitness norms such as those published by the American College of Sports Medicine. Nevertheless, the difference between these two approaches to setting norms should be relatively small since it is known that the physical fitness of Taiwanese adolescents is comparable to adolescents from other countries, as least those in the Asian region^37^.

Last but not least, there were numbers of factors relating to academic performance, such as cognitive function, intelligence, after-curriculum physical activity, presence of psychological or neurological disorders, or individuals with special education needs that were not considered by the current study. It is possible that the present results were confounded by these factors and future research is needed to investigate these factors further.

In conclusion, the strengths of the current study include its use of a wide range of measures of physical fitness, a large nationally representative sample, and the use of a well standardized measure of academic measure. The major findings of this study are that: (a) levels of aerobic fitness were found to be most closely related to academic performance relative to muscular strength, endurance and flexibility; and (b) for each fitness component, students who maintained high levels of fitness (i.e. within the top 25% PR) throughout the 3 years of junior high school outperformed all other groups in academic performance to a degree likely to make a practical difference to the quality of high school that they will be invited to attend. It is recommended that future research extend the results of the present study by employing interventional designs that manipulate exercise over time, and study the effects between different types of fitness on academic achievement while controlling for potential confounders (e.g., socioeconomic status, psychological or neurological disorders, and intelligence).

The current findings imply that there are academic advantages of being highly fit in the first year of high school (perhaps as a consequence of fitness at the end of elementary school) and maintaining this fitness throughout the three years of junior high school. If a high level of fitness is not possible to achieve in year 1, it there are still academic advantages to be had from improving fitness to a high level by year 3. This is particularly true in the case of aerobic fitness. The physical activity needed to achieve this fitness can occur at any time that would not compete with time spent on academic instructions. With this in mind, public policy initiatives are needed to support programs to increase physical activity in primary and middle school that in turn will foster learning and academic achievements in the long term.

## Data Availability

The datasets generated and/or analyzed during the current study are available from the corresponding author on reasonable request.
